# Context changes retrieval of prospective outcomes during decision deliberation

**DOI:** 10.1093/cercor/bhae483

**Published:** 2024-12-22

**Authors:** Pinar Göktepe-Kavis, Florence M Aellen, Aurelio Cortese, Giuseppe Castegnetti, Benedetto de Martino, Athina Tzovara

**Affiliations:** Institute of Computer Science, University of Bern, 3012 Bern, Switzerland; Center for Experimental Neurology - Sleep Wake Epilepsy Center - NeuroTec, Department of Neurology, Inselspital Bern, University Hospital, University of Bern, 3010 Bern, Switzerland; Institute of Computer Science, University of Bern, 3012 Bern, Switzerland; Center for Experimental Neurology - Sleep Wake Epilepsy Center - NeuroTec, Department of Neurology, Inselspital Bern, University Hospital, University of Bern, 3010 Bern, Switzerland; Computational Neuroscience Laboratories, Advanced Telecommunications Research Institute International, 619-0288 Kyoto, Japan; Institute of Cognitive Neuroscience, University College London, London WC1N 3AZ, United Kingdom; Institute of Cognitive Neuroscience, University College London, London WC1N 3AZ, United Kingdom; Institute of Computer Science, University of Bern, 3012 Bern, Switzerland; Center for Experimental Neurology - Sleep Wake Epilepsy Center - NeuroTec, Department of Neurology, Inselspital Bern, University Hospital, University of Bern, 3010 Bern, Switzerland

**Keywords:** context, decision-making, EEG, multivariate decoding, outcome retrieval

## Abstract

Foreseeing the future outcomes is the art of decision-making. Substantial evidence shows that, during choice deliberation, the brain can retrieve prospective decision outcomes. However, decisions are seldom made in a vacuum. Context carries information that can radically affect the outcomes of a choice. Nevertheless, most investigations of retrieval processes examined decisions in isolation, disregarding the context in which they occur. Here, we studied how context shapes prospective outcome retrieval during deliberation. We designed a decision-making task where participants were presented with object–context pairs and made decisions which led to a certain outcome. We show during deliberation, likely outcomes were retrieved in transient patterns of neural activity, as early as 3 s before participants decided. The strength of prospective outcome retrieval explains participants’ behavioral efficiency, but only when context affects the decision outcome. Our results suggest context imparts strong constraints on retrieval processes and how neural representations are shaped during decision-making.

## Introduction

In our everyday lives, we make numerous decisions that are prompted and affected by the context around us. For example, choosing whether to carry an umbrella with us largely depends on the season we are in and the weather forecast. While we make choices, our brains engage in prospective planning, where the possible outcomes of our actions are retrieved and reinstated at the neural level (Redish 2016; Rich and Wallis 2016; Shadlen and Shohamy 2016; Castegnetti et al. 2020; Crivelli-Decker et al. 2023; Wimmer et al. 2023). Retrieval of prospective outcomes is considered an integral ingredient of decision-making across many species: for example, in rodents, future trajectories of choices are reinstated in the hippocampus to assist in goal-oriented navigation (Pfeiffer and Foster 2013; Ólafsdóttir et al. 2018). Similarly, the human brain represents possible future outcomes and states of the environment during decision deliberation (Castegnetti et al. 2020; Wise et al. 2021) and offline, during rest (Kurth-Nelson et al. 2016; Schuck and Niv 2019). A number of benefits have been suggested for the reinstatement of neural representation of previously experienced or prospective information. This includes supporting the consolidation of learned information (Schapiro et al. 2018) and planning (Singer et al. 2013; Schuck and Niv 2019; Eldar et al. 2020; Wimmer et al. 2023). In short, neural retrieval of outcomes is critical to facilitate learning to achieve future goals (Mattar and Daw 2018; Liu et al. 2021; Wise et al. 2021).

Although retrieval of neural representations has been observed across tasks and species, circumstances under which neural representations are retrieved in the brain is an open question. Recent computational frameworks have proposed that neural retrieval during deliberation manifests depending on the combination of how often an option is expected to be chosen and the expected reward gain after choosing an option (Mattar and Daw 2018). However, such neural calculations underlying deliberation are computationally expensive and may therefore be omitted in decisions that do not require strenuous deliberation (Keramati et al. 2011; Mattar and Daw 2018). Although such decisions are rather fast, they are not sensitive to the changes in the environment such as context or goal switches (Keramati et al. 2011). On the other hand, the behavioral readout of the decisions that need deliberation demonstrates higher flexibility at the cost of longer reaction times (Daw et al. 2005; Balleine and O’Doherty 2010; Keramati et al. 2011). This trade-off leads to both types of decisions to occur, depending on deliberation demands (Daw et al. 2005; Balleine and O’Doherty 2010; Keramati et al. 2011; Dolan and Dayan 2013). Several factors modulate the demands for deliberation, including whether a given decision is goal-oriented (Dolan and Dayan 2013), its difficult,y, or whether the action-outcome associations are well-learned (Tzovara et al. 2015). Although prospective outcome retrieval is considered to largely benefit and underlie decision-making, it remains unknown whether and how it manifests as a function of deliberation demands.

To address this question, we designed a novel experimental protocol, where the need for choice deliberation is modulated by the context into which a decision takes place: deliberation about whether to carry an umbrella or not may be beneficial in spring, where the weather is more changeable, but less so in the middle of the summer or during winter. Converging evidence suggests that context is an integral part of decision-making, acting on multiple levels, from sensory representations to abstract valuation (Grueschow et al. 2015; Frömer et al. 2019; Castegnetti et al. 2021; Moneta et al. 2023; Schaffner et al. 2023), shaping neural representations and attentional allocation (Grueschow et al. 2015; Frömer et al. 2019; Sepulveda et al. 2020; Castegnetti et al. 2021; Moneta et al. 2023; Schaffner et al. 2023). The context into which a decision is made plays a crucial role in identifying the desired choice (Sepulveda et al. 2020; Moneta et al. 2023), suggesting that context is inherently integrated into the deliberation process. However, most of existing studies used functional magnetic resonance imaging which has a very low temporal resolution, making it difficult to determine at what point during deliberation the context is integrated. At the same time, most studies on outcome retrieval while using techniques with a better temporal resolution (e.g. intracranial recordings, electroencephalography [EEG], or magnetoencephalography [MEG]) have often treated decisions detached from context. These leave 2 important questions unanswered: (i) how context affects the neural computations underlying decision deliberation and (ii) how early in time it is integrated into the decision-making process.

This work aims to bridge the gap between these 2 strands of research. We investigated how the neural representation of likely decision outcomes unfolds over time before a choice is made, and how retrieval of these representations is altered by context. To achieve this, we employed high-density scalp EEG together with multivariate decoding to detect the neural representation and retrieval of prospective outcomes during deliberation. We hypothesized that outcome representations are retrieved in the brain while deliberating and that outcome retrieval would be stronger and behaviorally relevant for those decisions where context determines their outcome. By examining the interplay between context and the neural retrieval of prospective outcomes, our study aims to provide new insights into the complex neural mechanisms underlying decision-making and how they are modulated by contextual factors.

## Materials and methods

### Participants

Thirty healthy volunteers (16 women and 14 men, mean age 24.9 ± 4.2 SD) participated in the study. The sample size was selected following common practices in recent studies using similar techniques as ours to study the neural retrieval of outcome in decision-making (Castegnetti et al. 2020; McFadyen et al. 2023; Wimmer et al. 2023). The experiment protocol was approved by the Health, Social, and Cantonal Ethics Committee of the canton of Bern, Switzerland (reference number: 2020-00060). All participants reported having a normal or corrected-to-normal vision. Before the experiment, a written informed consent was signed by the participants.

### Experimental procedures

**Fig. 1:**
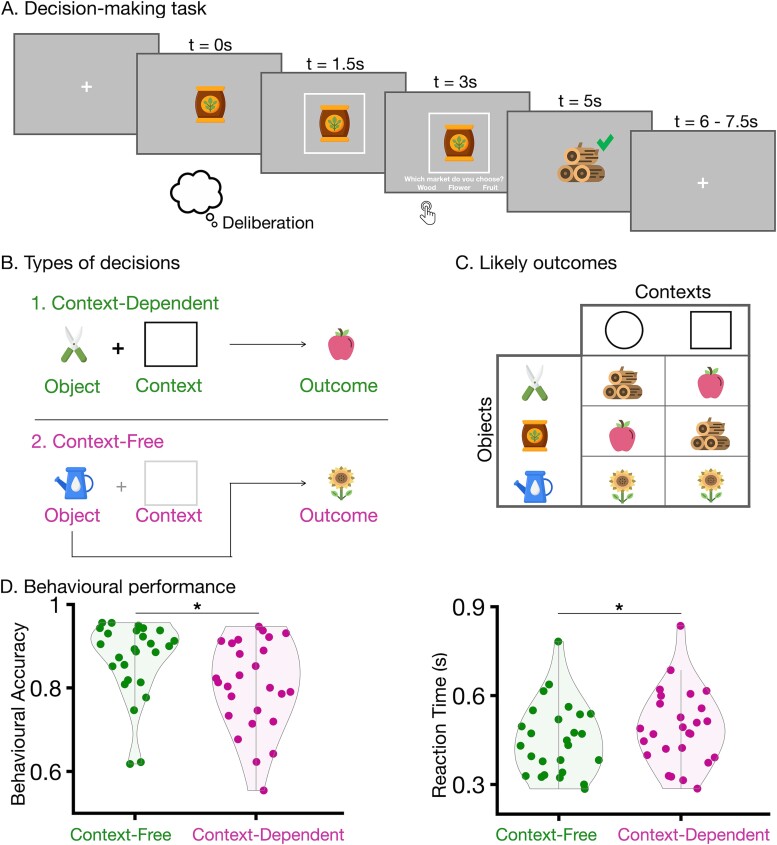
Task design and behavioral outcomes. A) Design decision-making task. Each trial started with an object (gardening tool) which was presented for 1.5 s. then the context (fictional season) was presented together with the object for 1.5 s. This period of the first 3 s was considered as decision deliberation. Next, participant’s decisions on which market to sell the gardening outcome given the object and context were asked. After the participant’s response, the outcome was presented together with a sign (tick or cross) for 1 s and followed by a 1.5 to 2.5 s long fixation cross. The thought balloon indicates the deliberation period before a decision was made. B) The types of decisions in the task. A decision was context-dependent if object and context together determined the outcome. It was a context-free decision when only object was enough to determine the decision outcome. C) Exemplar object-context-outcome association table. Three gardening tools (the most left column) were used as objects and 2 geometric shapes (top row) indicated the fictional seasons. Every object-context pair was associated with a gardening outcome. For 2 objects, context determined the likely outcome of a given object (first 2 rows of the table) whereas for the control condition outcome was independent of context (last row of the table). d) Behavioral accuracy in context-free was significantly higher than in context-dependent decisions [Wilcoxon signed-rank test, z(25) = 3.14, *P* < 0.002]. e) RT for context-dependent decisions was significantly longer than for context-free decisions [Wilcoxon signed-rank test, z(25) = −3.42, *P* < 0.001]. In B and C, each dot corresponds to a participant’s average behavioral accuracy in context-free or context-dependent decisions.

The experimental procedure consisted of 2 phases: a functional localizer, and the main decision-making task. As stimuli, a set of images with a gardening theme was used (Fig. 1C). Each stimulus was presented at the center of the screen in order to minimize eye artifacts in EEG responses. Stimuli were delivered by the PsychoPy package (version: 3.1.5) in Python (version: 3.7.3), on a monitor positioned 75 cm away from participant’s forehead, with a visual angle of 6° 15′ 0.49″.

### Functional localizer

Before the main decision-making task, participants took part in a functional localizer. The goal of the localizer was to isolate EEG responses to 3 outcome stimuli from confounding neural processes that would be present in the decision-making task, such as motor responses or decision-related variables. Participants were presented with an outcome image for 0.3 s, followed by a fixation cross, presented for 1.5–2.5 s (Fig. S1). To ensure that participants were paying attention to the localizer, they were asked to fixate on the presented images and press a button every time that the fixation cross changed color from white to red. The cross was white in 90% of the trials and red in 10%. All participants were able to detect all the red crosses with an average response time (RT) of 0.47 ± 0.01 s. This session consisted of 100 presentations of each outcome image, or 300 trials in total.

### Decision-making task

We designed a novel decision-making task to investigate how the neural retrieval of outcomes during decision deliberation is affected by the decision context (Fig. 1A). The task followed a narrative according to which participants role-played a gardener. On each trial, they were presented with a gardening tool (object, which could be scissors, watering can or fertilizer) and a fictional season (context, highlighted by a rectangle or circle surrounding the object, Fig. 1A). On each trial, participants were asked to make a decision on the market to which they would sell the gardening outcome of the presented object-context pair in order to maximize their profit. The possible markets (wood, flower, fruit) were revealed 1.5 s after the presentation of the context, prompting participants to select 1 of the 3 via a button press. In the narrative of the task, the gardener could only sell the product if it matched the market. Otherwise, they would lose money as the product would not be suitable for the chosen market and would be lost due to rotting. Participants indicated their responses through a button press on a keyboard.

Every pair of object and context was associated with one most likely outcome with an 80% probability, and the other 2 outcomes with a 10% probability each (Fig. 1C). In the majority of decisions (320 trials; 67% of all decisions), context determined the likely outcome of a given object (context-dependent), while in the control condition outcome was independent of context (context-free; Fig. 1B). An exemplar object-context-likely outcome association table is provided in Fig. 1C. These associations were randomized across participants but kept constant throughout the experiment for a given participant.

The task had the following structure (Fig. 1A, lower panel): first, an object was presented for 1.5 s and then the context was shown together with the object for 1.5 s. Next, the 3 possible markets were revealed prompting participants to select one. After participants made their choice, there was a 0.5 s wait period and subsequently their chosen outcome was presented together with feedback on whether the decision was correct (green tick) or wrong (red cross).

EEG recordings started with the functional localizer (~9 min), and then participants received a briefing about the decision-making task. This task consisted of 8 sessions, each of which contained 10 presentations of every object-context pair, the decision for this pair, and the outcome (lasting 6–7.5 min), resulting in 480 decision-making trials.

### Analysis of behavioral readouts

Participants’ behavioral performance was assessed by the percentage of trials in which they selected the most profitable market, given the presented object-context pair and also by their RT. To test the effect of context on behavioral performance, non-parametric paired Wilcoxon signed-rank tests were applied to statistically compare the average behavioral performance and RTs across participants for context-free vs. context-dependent decisions.

### E‌EG recordings and preprocessing

EEG data were recorded with a 256-channel Geodesic sensor system (Electrical Geodesics Inc.) with 1,000 Hz sampling rate and an online reference at the Cz electrode. After the acquisition, raw EEG data were filtered between 1–20 Hz. Among the available channels, 48 horizontal ocular and cheek channels were excluded from the EEG analysis due to persistent muscle artifacts, resulting in 208 electrodes. Heartbeat, eye blink, and eye movement artifacts were removed by independent component analysis (Hyvarinen 1999) followed by manual inspection. For the functional localizer, trials were extracted from −0.1 to 1 s relative to stimuli presentation. From the main task, we extracted 3 types of trials: outcome trials (−0.1 to 1 s relative to outcome onset); deliberation trials −0.1 to 1 s relative to object onset and − 0.1 to 1 s relative to context onset. Baseline correction was not applied to avoid contamination of outcome and deliberation periods. Noisy epochs affected by muscle activity, movement or other artifacts that remained after independent component analysis were excluded based on visual inspection of the data. This resulted in the exclusion of on average 22% of the epochs (mean 66.7 ± 23.6 SD). Noisy electrodes were interpolated (on average 14.6% across participants). After data cleaning, epochs were re-referenced to the common average reference and down-sampled to 256 Hz, to reduce computational time for the decoding analysis, as it is commonly done in the field (Liu et al. 2019; Castegnetti et al. 2020; Wise et al. 2021). Data preprocessing was done using MNE library (version 0.24; Gramfort et al. 2013). Two of the participants were excluded from the analyses because of large artifacts in their EEG signals that could not be cleaned. Two additional participants failed at learning the task and were therefore also excluded. Hence, the final dataset that was used in all analyses contained 26 participants.

### Multivariate decoding analysis

**Fig. 2:**
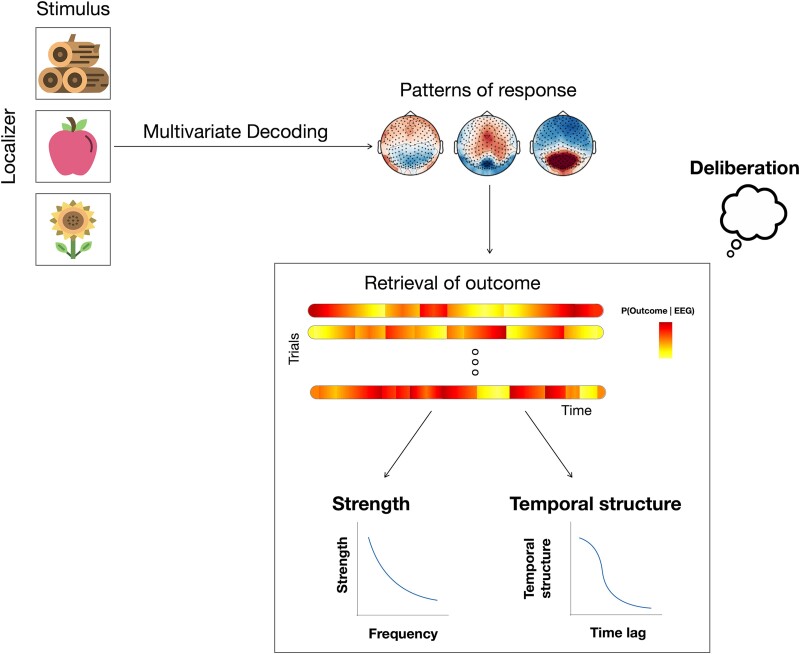
Schematic representation of our data analysis. Top: patterns for the three outcome images were decoded by training classifiers on EEG responses during the functional localizer, as the first step of the analysis. Bottom: the decoded and tested patterns were then used to detect outcome representation during deliberation, before making a decision. We first reconstructed the probability of outcome representations at every time point on the single-trial EEG responses during deliberation based on the classifiers trained to discriminate the three outcomes. As an outcome can be retrieved at any time during deliberation, we analyzed the strength and temporal structure in retrieval of outcome representations via PSD and autocorrelation of reconstructed probabilities on single-trial level.

In order to extract EEG patterns reflecting neural responses to the identity of the 3 possible outcomes, we trained classifiers on the EEG responses to the 3 outcome images presented during the functional localizer (Fig. 2). Prior to the decoding analysis, EEG data were normalized to zero mean and unit variance, as is common practice in the field. We used logistic regression to train and test one 3-class classifier per time point across the trial and participant, in a 5-fold cross-validation, using L2 regularization. Decoding performance was quantified via the classification accuracy (ratio of correctly classified trials along the 3 classes). In a control analysis, the same approach was followed to decode the identity of the 3 outcomes during the decision-making task by using EEG responses during the outcome presentation (Fig. S2a) after a decision was made. Last, we tested the generalization of the classification models trained on EEG responses in the functional localizer and tested on the EEG responses to outcomes during the decision-making task (Fig. S2b). In all cases, we performed the decoding analysis at the single-participant level and then averaged the time-courses of classification accuracy across the group of participants. For the classification analysis, we used MNE (Gramfort et al. 2013) and Scikit-Learn (Pedregosa et al. 2011).

### Estimation of chance levels

Chance was computed empirically for each participant by training classifiers using the same training data, but randomly permuting their labels 100 times. To identify time periods with above-chance decoding performance, the decoding performance of “true” classifiers was statistically compared to empirical chance levels by non-parametric Wilcoxon signed-rank tests and corrected for multiple comparisons across time via the false discovery rate (FDR).

### Reconstruction of outcome representation during deliberation

**Fig. 3:**
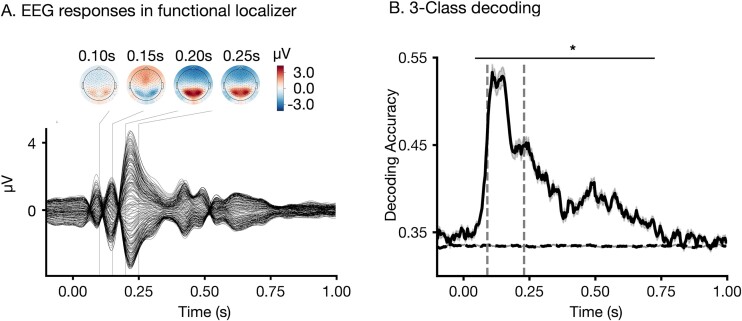
EEG responses to outcomes and decoding of outcome identity from EEG responses. A) Average EEG responses to outcome images presented during the functional localizer across the group of participants. B) Time point-by-time point decoding of the identity of the three outcomes. Black solid line shows the group average of decoding accuracy whereas black dashed line shows the chance level. Gray-shaded areas show standard error. Horizontal bar with asterisks (*) indicates time periods in which decoding accuracy was above chance. Gray dashed vertical lines at 0.09 and 0.23 s show the time period used for training final decoders which were used to detect outcome representations during deliberation.

To study the neural retrieval of prospective outcome we used the decoding models trained on the EEG responses to the functional localizer and applied them in the deliberation phase of the main experiment, before the outcome was revealed. To this aim, we used EEG responses to the localizer between 0.09 and 0.23 s post-stimulus onset, to train 1 model per participant, reflecting the patterns of their EEG responses to each of the 3 possible outcomes. This window was selected as it corresponded to the temporal interval where decoding performance in the localizer was the strongest (Fig. 3B). We opted for using a time window instead of a fixed time point to account for inter-individual variability and to capture more diverse patterns of outcome representation, following a similar approach as previous studies (Wise et al. 2021). Using this temporal window, we trained 1 classifier per participant using all trials from the localizer to represent neural patterns corresponding to the 3 possible outcomes (Fig. 2 top row). As these classifiers were trained using data of the localizer, isolated from the decision-making task, they solely represent neural activity related to the identity of the 3 possible outcomes (neural representation of outcomes), dissociated from confounding factors like valuation, decision-making, or motor response. The trained classifier for each participant was then applied on EEG data recorded during the decision-making task, in the deliberation period, which corresponded to the time period before an outcome was chosen. As this time period is unlabeled (i.e. there are no actual outcome images presented), we computed the probability of a given outcome to be represented given the EEG data, using the following formula:P (Outcome|EEG_t_) = 


(1) \begin{equation*} \frac{1}{1+{e}^{-\left({\beta}_0+{\beta}_1{EEG}_{t,1}+\dots +{\beta}_n{EEG}_{t,n}\right)}} \end{equation*}

where ${\beta}_n$ are the classifier weights and ${EEG}_{t,n}$ the EEG measurements recorded from channel $n$, with *n* = 208 channels in total, at time point t. This corresponds to the step before assigning the labels. Outcome probabilities were computed for each time point (t) and trial. In our analyses we focused on the probabilities of the most likely outcome out of 3 possible outcomes (Fig. S3 for exemplar time course of reconstructed probabilities). For context-dependent decisions, 2 outcomes were equally likely before the presentation of context. Therefore, for this case we reconstructed probabilities for both outcomes separately. Since we were interested in the likely outcome, for or time-locked analysis, when studying the effect of context, the probability values were binarized by applying a threshold of 0.33 to the reconstructed probabilities. The resulting values reflected whether the likely outcome was “represented” or not. The reported results remained similar whether we binarized or not. All other analyses were conducted on the actual probabilities without binarizing. The time-locked results were also replicated without binarizing.

### Quantifying outcome retrieval during deliberation

**Fig. 4:**
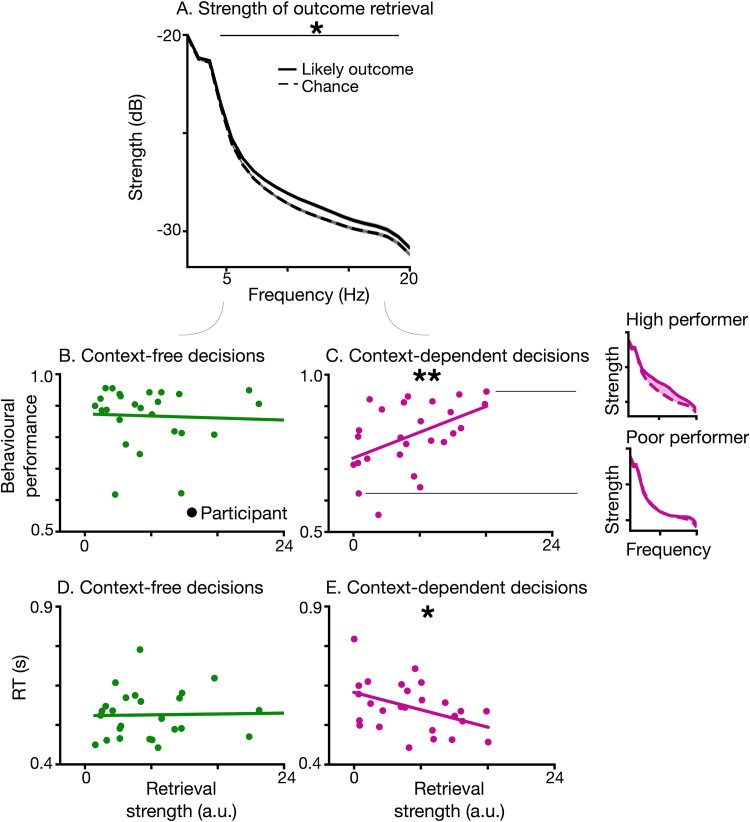
Outcome retrieval and its relation with behavioral performance. A) Strength of outcome retrieval (solid black line) vs. chance (dashed line), quantified via power spectral analysis on reconstructed outcome probabilities. Chance level was evaluated based on the randomly permuted classifiers of outcome images. Horizontal bar with asterisks (*) indicate frequencies which were different from chance. B and C) Neuro–behavioral coupling between the behavioral performance and strength of outcome retrieval. Regression of behavioral performance on strength of outcome retrieval in B) context-free and V) context-dependent conditions. In context-free decisions, there was no significant regression of behavioral performance on strength of outcome retrieval [panel B, linear regression, F(1, 24) = 0.08, *P* = 0.78]. However, the regression was significant for context-dependent decisions [panel C, linear regression, F(1, 24) = 6.98, *P* = 0.014]. For context-dependent decisions (panel C), single-subject results are provided for 2 exemplar participants (1 with high behavioral performance, and another 1 with low behavioral performance). D and E) Neuro–behavioral coupling between the RTs and strength of outcome retrieval for D) context-free and E) context-dependent decisions. In context-free decisions, there was no significant regression of RT on the strength of outcome retrieval [panel D, linear regression, F(1,24) = 0.022, *P* = 0.88], which was the case for context-dependent ones [panel E, linear regression, F(1,24) = 5.39, *P* = 0.03].

As outcome representations can be retrieved at any time point during deliberation, we quantified outcome retrieval via the strength and temporal structure of single-trial reconstructed probabilities via the power spectral density (PSD; Fig. 4) and autocorrelation function (Fig. S4), respectively. The PSD was used to uncover the strength of patterns of reconstructed probabilities that were not necessarily time-locked to external events, while the autocorrelation function was used to quantify the temporal structure of outcome retrieval. To ensure that our results were not driven by properties of EEG signals like a power law but reflected outcome probabilities, we computed the same metrics but based on the randomly trained classifiers, which provide a measure of chance, and then computed the area under the curve between PSDs and temporal autocorrelation obtained by real vs. chance probabilities.

We computed the strength of prospective outcome retrieval by extracting the single-trial power of the time course of reconstructed outcome probabilities [P (Outcome|EEG_t_)]. We used multitapers, applied on the single-trial time-course of reconstructed probabilities in the frequency range of 1 to 20 Hz (using time_frequency.psd_array_multitaper function from MNE with adaptive weights and full normalization). The resulting power spectra were then averaged across deliberation trials. For context-dependent decisions, before context was revealed, there were 2 equally likely outcomes. Therefore, PSDs were computed for each likely outcome separately and then averaged together.

We quantified the single-trial temporal structure of reconstructed outcome probabilities by computing their temporal autocorrelation, which quantifies how similar a time-series signal is to its lagged (future or past) values. The autocorrelation was computed for time-lags up to 0.3 s, with steps of 0.004 s, based on single-trial probabilities, and was then averaged across trials, similar as in previous studies (Castegnetti et al. 2020).

To test whether neural representation of prospective outcome was retrieved during the deliberation period, we contrasted the PSD and autocorrelation of the likely outcome probabilities to chance levels. We estimated chance levels via the decoding models obtained by randomly permuting the classifier labels and repeating the calculation of PSD and autocorrelation based on these chance models. The PSD and autocorrelation of a given outcome were then compared to that of random permutations using Wilcoxon signed-rank test and were corrected for multiple comparisons over frequencies (for the PSD) and time-lags (for the autocorrelation) via FDR correction.

### Neural–behavioral coupling

To study the link between neural retrieval of prospective outcome and behavioral outputs, we quantified for each participant the strength and temporal structure of outcome retrieval and then tested its links to behavioral performance and RTs. We computed for each participant the average PSD or autocorrelation across the relevant deliberation trials and the computed the area between the PSD corresponding to probabilities of the true and chance decoding models (Fig. 4C, side panels for exemplar participants). We focused on those frequencies (PSD) where the group-level measures were different from chance in the condition of interest. These were chosen for 2 reasons: first because these frequencies reflect unambiguous signatures of prospective outcome retrieval, where it is represented above chance levels, and second because this allowed us to keep the selection of frequencies orthogonal to the neural–behavioral coupling analysis. The outcome retrieval strength across participants was then regressed on their behavioral performance and RTs with linear regression. We used a regression model that included 1 factor for outcome retrieval (strength or temporal structure), and 1 factor for context-dependence (context-free vs. context-dependent decisions) as well as their interaction.

We further investigated the observed link between outcome retrieval and behavior by analyzing the time course of outcome retrieval strength and behavioral performance (Fig. S5 for group-level average behavioral performance over the course of experiment) throughout the experiment on a single-participant level. To this aim, we computed the time course of outcome retrieval by calculating the area between average PSD corresponding to probabilities of the true decoding models and chance models over sub-averages of *n* = 20 trials per object and context pair for each participant. The behavioral accuracy of respective trials was also averaged. We repeated this process in a sliding window fashion, resulting in a time course of outcome retrieval strength and behavioral accuracy. Employing an approach previously used to detect changes in learning rate (Frank et al. 2022) and behavioral sensitivity (Tom et al. 2007), we quantified changes in behavior as well as outcome retrieval over the course of the experiment by fitting a linear model across trials (Fig. S6 for exemplar time-course of behavioral accuracy with fitted models). Then we computed the speed of change in behavioral accuracy and in retrieval strength by extracting the slope of a linear model, fitted to every 10 trials (this analysis was repeated with *n* = 5 and *n* = 15 showing similar results). With this analysis we aimed at testing the hypothesis that an improvement in behavioral performance would be explained by a change in outcome retrieval. For this reason, we identified positive model fits (slopes) for behavioral changes and slopes of the outcome retrieval in the respective trials. All positive slopes of behavioral accuracy were then averaged per object and context pair in context-free and context-dependent decisions for each participant as an aggregate indicator of learning speed. We averaged all the slopes of outcome retrieval that correspond to positive slopes of behavior. This analysis was performed separately for each context/object/outcome association, as each of those could be learned at a different speed. We then regressed the improvement in behavior to the absolute change in outcome retrieval strength with a linear mixed effects model with a random factor of participants.

## Results

Thirty healthy participants performed a decision-making task while their neural activity was being recorded via high-density EEG. In our task participants were instructed to play the role of a virtual market farmer. They had to select the market they would sell their product to. Each participant was shown fictional gardening tools (object) and seasons (context; Fig. 1A) for 1.5 s. They learned outcome-object-context associations by trial and error. Each object-context pair was associated with one most likely outcome with 80% probability (Fig. 1C). For 2 out of 3 objects, their likely outcome was determined by the context (context-dependent, Fig. 1B). For the third, control object, the outcome was independent of context (context-free, Fig. 1B). Prior to the main task, participants took part in a localizer, where they were exposed to the 3 possible outcomes, presented in a random order (Fig. S1).

Participants were significantly better at selecting the market to which they could sell the corresponding gardening outcome, which had to match with the market to be sold, in context-free (likelihood of selecting likely market: 0.87 ± 0.02 mean ± SEM here and in the following) than in context-dependent (0.81 ± 0.02) decisions [Wilcoxon signed-rank test, z (25) = 3.14, *P* < 0.002]. They were also significantly faster in indicating their choice in context-free (0.45 s ± 0.02 s) than in context-dependent (0.49 s ± 0.02 s) decisions [Wilcoxon signed-rank test, z (25) = −3.42, *P* < 0.001]. These results provide a first indication that additional neural processes or computations may take place when information about the context must be integrated and used to guide a decision.

### Decoding outcomes from EEG activity patterns

To investigate whether decision outcome is represented the neural level during decision deliberation we used multivariate pattern analysis (Fig. 2, top; Castegnetti et al. 2020; Eldar et al. 2020; Wise et al. 2021). We focused on EEG responses to the three possible outcomes recorded in the pre-task functional localizer session to obtain neural representations of the possible decision outcomes unconfounded by decision-making or contextual variables (Fig. 3A). Multivariate decoders were trained to discriminate EEG responses to the three possible outcomes for each participant (Fig. 2, top). Classification performance was above chance (*P*_FDR_ < 0.05) from 0.05 to 0.72 s post-stimulus onset and peaked at 0.11 s with a 3-class mean accuracy of 0.53 ± 0.01 across participants (Fig. 3B). Chance level decoding performance was estimated by training 100 classifiers on randomly permuted labels, and was around 0.33, which corresponded to the theoretical chance level (Fig. 3B, dashed line).

After training these classifiers, we ensured that decoding during the outcome presentation was above chance (Fig. S2a) and that trained classifiers generalized from the pre-task localizer to the main task (Fig. S2b). We then proceeded with studying how the neural retrieval of outcome unfold in a prospective manner, and what their behavioral relevance is. To this aim, we retrained 1 outcome decoder per subject, over a time window of 0.09–0.23 s post-stimulus onset, using the localizer EEG responses. This temporal window was chosen because it exhibited the strongest decoding performance and covered the peak in the decoding analysis in the functional localizer decoding (Fig. 3B), outcome presentation (Fig. S2a), as well as in the localizer-to-outcome generalization (Fig. S2b).

### Neural representation of prospective outcome

Next, we focused on the decision deliberation period (Fig. 1A) and we examined whether the prospective outcome is represented in the brain. To test this, we computed the probability of the most likely outcome to be represented on single-trial EEG activity during decision deliberation (Fig. 2, bottom) before and after presentation of context separately. We assessed whether neural representations of outcome were retrieved during decision deliberation by computing the strength of reconstructed probabilities, assuming that outcome can be retrieved at any time point during deliberation, and not in a strictly time-locked manner.

To compute the strength of outcome retrieval, we calculated the PSD of reconstructed probabilities of the most likely outcome for each individual decision before and after context presentation separately. In context-dependent decisions during the period before context presentation, 2 outcomes were equally likely. Therefore, in this condition we performed our analysis for each outcome separately and then averaged the resulting power spectra. Then we compared that to the PSD of probabilities obtained via randomly trained classifiers for the likely outcome, which quantified chance (Fig. 4A). We found a significantly stronger than chance power in the decoded outcomes during decision deliberation between 4.5 and 19.1 Hz (Wilcoxon signed-rank test, *P*_FDR_ < 0.05, Fig. 4A, see Fig. S7 for PSDs of outcome retrieval and of EEG data and Fig. S8 for control analysis of oscillatory activity in EEG). Stronger than chance power in outcome representations suggests that prospective outcome was retrieved in the brain during decision deliberation. This finding was also confirmed by computing the temporal autocorrelation of reconstructed outcome (Fig. S4a). As outcome retrieval deviated from chance at certain frequency range and time lags, which shows that retrieval of outcome is a structured process during deliberation. These findings support our hypothesis that likely outcomes are retrieved at the neural level during decision deliberation, before the outcome itself is experienced.

### Neural–behavioral coupling during choice deliberation

After establishing that outcome is retrieved during deliberation, we next tested how its retrieval is affected by deliberation demands, and whether it is related to behavioral readouts. For each participant, we quantified the strength and temporal structure of outcome retrieval during deliberation before and after context presentation as the area between likely outcome and chance curves in their power spectra (Fig. 4, exemplar single-subject results in bottom panels) and temporal autocorrelation, respectively. Then, we computed a regression of the behavioral decision accuracy of each participant on strength of outcome retrieval during deliberation, including both before and after context periods.

To this aim, we expressed behavioral performance as a function of the strength of prospective outcome retrieval, of context-dependence, and their interaction. The resulting model [F(3, 48) = 4.26, *P* = 0.01] revealed a significant interaction between the strength of prospective outcome retrieval and context (*P* = 0.03), and a main effect of context relevance (*P* = 0.003), while the strength of outcome retrieval alone was not significant (*P* = 0.78). In a post-hoc analysis, we regressed behavioral performance on outcome retrieval separately for context-free and context-dependent decisions. For context-dependent decisions, we found a significant regression of behavioral performance on the strength of outcome retrieval [F(1, 24) = 6.98, *P* = 0.014, Fig. 4C]. We found largely similar results when repeating this analysis on intervals before [F(1,24) = 7.5, *P* = 0.011] and after [F(1,24) = 5.39, *P* = 0.03] context presentation separately (Fig. S9). For context-free decisions there was no significant relationship between behavioral performance and strength of outcome retrieval [F(1, 24) = 0.08 *P* = 0.78, Fig. 4B; while a complementary analysis based on the temporal autocorrelation can be found in Fig. S4]. Moreover, there was a significantly negative regression of RTs on outcome retrieval for context-dependent [F(1,24) = 5.39, *P* = 0.03, Fig. 4E] but not for context-free decisions [F(1,24) = 0.022, *P* = 0.88, Fig. 4D]. These results show that in context-dependent decisions there is a strong neuro–behavioral coupling between prospective outcome retrieval and choice: participants with stronger retrieval of prospective outcome (Fig. 4C) were the ones more likely to select the likely outcome and did so faster. By contrast, in context-free decisions the strength of outcome retrieval was not related to behavioral performance and RT.

To investigate whether this neuro–behavioral coupling was specific to the retrieval of the most likely outcome in the deliberation phase we performed 2 additional analyses. First, we tested that the neuro–behavioral coupling was not trivially driven by the decoding models that discriminate the three outcomes. To this aim, we repeated the regression analysis but this time between behavioral performance and accuracy of the classifiers that discriminate EEG responses to three outcomes, based on the pre-task localizer. We found no significant regression of behavioral performance on outcome classification performance neither for context-free [Fig. S10, left panel, green, linear regression, F(1, 24) = 0.07, *P* = 0.8] nor context-dependent decisions [Fig. S10, right panel, pink, linear regression, F(1, 24) = 0.05, *P* = 0.83].

Second, we ensured that the neuro–behavioral coupling in the context-dependent decisions and its lack in context-free ones was not due to a lack of behavioral variability in the latter. To exclude this possibility, we repeated this analysis for the early and late phases of the experiment, which corresponded to periods of high vs. low behavioral variability across participants (Fig. S11). We found a significant and strong regression of behavioral performance on the strength of prospective outcome for both early [Fig. S11b, left, pink, F(1, 24) = 4.94, *P* = 0.036] and late [Fig. S11b, right, pink, F(1, 24) = 5.40, *P* = 0.029] context-dependent decisions. For context-free decisions, there was no significant regression neither for early [Fig. S11b, left, green, F(1, 24) = 1.23, *P* = 0.28] nor for late decisions [Fig. S11 right, green, F(1, 24) = 0.16, *P* = 0.69], although the former were characterized by strong behavioral variability. These analyses suggest that the neural–behavioral coupling is specific to context-dependent decisions and reflects decision deliberation and not the quality of EEG decoding or variability in behavioral performance.

We further investigated the observed coupling between outcome retrieval and behavior by analyzing the time-course of retrieval strength and behavior over the time-course of the experiment. With this analysis we quantified whether the speed of learning each specific object-context pair was related to changes in outcome retrieval and was thus performed for each context-free and context-dependent context/object pair separately (*n* = 2 for context-free and *n* = 4 for context dependent pairs for each participant). Across-participant differences in the overall speed of learning were adjusted with the use of mixed-effect models and a random factor for participants.

**Fig. 5:**
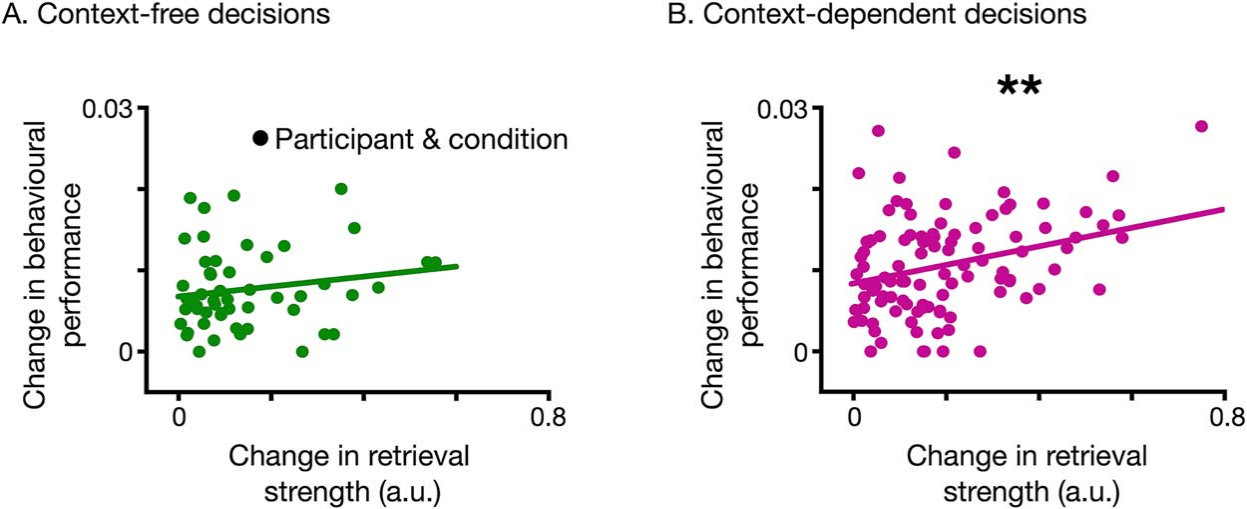
Neuro–behavioral coupling between the change in behavioral performance and in strength of outcome retrieval in A) context-free and B) context-dependent decisions. In context-free decisions, there was no significant regression of change in behavioral performance on change in retrieval strength [panel a, linear regression, F(1, 44.91) = 1.7, *P* = 0.2]. However, the regression was significant for context-dependent decisions [panel B, linear regression, F(1, 101.86) = 10.52, *P* = 0.0016]. Each data point corresponds to a pair of object and context for a participant. For visualization purposes the data points from each participant were not grouped together however, analysis was done with a linear mixed effect model, taking into account the participants as a random factor.

We expressed the improvement in behavioral performance (probability of selecting the most likely outcome over a time horizon of 10 outcomes) as the change in retrieval strength over the same time horizon of 10 decisions. We found a significant main effect of change in behavioral performance on change in retrieval strength for context-dependent decisions [F(1, 101.86) = 10.52, *P* = 0.0016, Fig. 5B] which remained significant when controlled for average behavioral performance (Fig. S12). However, for context-free decisions there was no significant relationship between changes in behavioral performance and changes in retrieval strength [F(1, 44.91) = 1.7, *P* = 0.2, Fig. 5A]. To control for different sample sizes in context-free and context-dependent associations, we did an analysis where we randomly excluded 2 object/context/outcome associations from context-dependent and repeated the regression analysis with a linear mixed-effects model. This procedure was repeated 100 times which resulted in significant regression (*P* < 0.05) in 71% of the times.

Overall, these results show that behavioral performance can be explained by the neural retrieval of outcome during decision deliberation, as a stronger outcome retrieval is found in better-performing participants in context-dependent, but not context-free decisions. This coupling between behavioral performance and retrieval strength was observed at the average level, across participants (Fig. 4) and also over the time-course of the experiment, as participants refined their internal models of context/object and outcome representations (Fig. 5), but again only in context-dependent decisions.

### Context alters decision deliberation

**Fig. 6:**
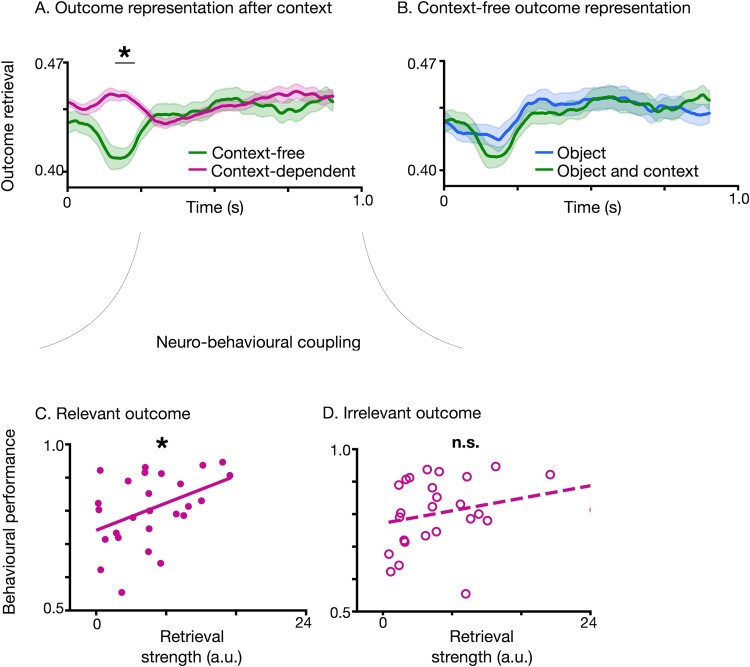
Effect of context on outcome retrieval. A) Time course of reconstructed outcome probabilities during decision deliberation, for context-free (green) vs. context-dependent (pink) decisions. Time zero corresponds to the time when context is revealed. Outcome retrieval was significantly stronger for context-dependent than context-free decisions, between 0.16 and 0.23 s post-context onset (Wilcoxon signed-rank test, *P*_uncorr_ < 0.05). B) Time course of reconstructed outcome probabilities, time-locked to the period when an object was presented alone without context (blue line) vs. the time period when the context was presented together with the object (green line), for context-free decisions. The time courses of reconstructed probabilities were identical, which suggests that for the case of context-free decisions, they were not affected by context. C and D) Neuro–behavioral coupling in context-dependent decisions after context presentation was significant for the c) relevant outcome [left panel, solid line, linear regression, F(1, 24) = 5.39, *P* = 0.03], but it was nonsignificant for the D) irrelevant outcome [right panel, dashed line, linear regression, F(1, 24) = 1.79, *P* = 0.19].

To evaluate the effect of context in decision-making further, we analyzed retrieval of prospective outcomes relative to the appearance of context in the decision-making task. Upon context presentation, the most likely outcome was more strongly retrieved for context-dependent decisions compared to context-free ones (Wilcoxon signed-rank test, *P*_uncorr_ < 0.05), starting at 0.16 s after context presentation (Fig. 6A, see Fig. S13 for reconstructed outcome probabilities without binarization). In the control, context-free condition, where context information was not needed for the decision, the retrieval of likely outcomes was not affected by the presentation of context, resulting in indistinguishable outcome retrieval probabilities before vs. after the presentation of context (Fig. 6B).

We next focused on context-dependent decisions, and on the time interval following context presentation. This is an important period, as it is the moment when the most likely outcome can be inferred given the presented context. We asked whether the neuro–behavioral coupling that we observed for context-dependent decisions was specific to the relevant outcome. We focused on context-dependent decisions, as they were the ones showing a neuro–behavioral coupling and computed the strength of retrieval of relevant (likely context-dependent) and irrelevant (context-free) outcomes (Fig. 6C and D). We then regressed behavioral accuracy on neural retrieval of relevant vs. irrelevant outcomes. We found a significant regression of behavioral performance on strength of relevant [Fig. 6C, F(1, 24) = 5.39, *P* = 0.03], but not irrelevant [Fig. 6D, F(1, 24) = 1.79, *P* = 0.19] outcomes. This finding suggests that the neuro–behavioral coupling that we observe in context-dependent decisions is specific to the neural retrieval of relevant outcome.

## Discussion

In this study, we investigated how contextual information is integrated in neural retrieval of prospective outcomes during deliberation by using a well-controlled new EEG task and multivariate decoding. We showed that the representation of prospective outcomes was retrieved by the brain during deliberation before the choice is made. Critically, we observed that the likely outcome was more strongly represented when context could alter the decision outcome, compared to when it was irrelevant, as early as 0.16 s after the presentation of context. This finding suggests that contextual information is integrated very early during outcome retrieval. Importantly, we found that during deliberation, outcome retrieval was mediating participants’ behavioral accuracy, but only when there was a clear deliberation demand to reach a decision. Our findings showed that decision deliberation involves neural retrieval of prospective outcome which reflects behavioral performance.

Previous work has shown that while making a choice our brains engage in retrieval of prospective outcome (Rich and Wallis 2016; Shadlen and Shohamy 2016; Castegnetti et al. 2020; Wimmer et al. 2023). This manifests in a variety of tasks as a mechanism used to consolidate learned information (Carr et al. 2011; Schapiro et al. 2018; Buch et al. 2021) and to reflect on prospective choices and planning mostly in navigation tasks by building and retrieving a cognitive map of task structure (Schuck and Niv 2019) to make memory-guided choices (Singer et al. 2013) which allows flexible adaptation to changing environments (Eldar et al. 2020; Wimmer et al. 2023). Our findings with a non-navigation task show that prospective outcome is represented in temporally structured patterns of neural activity. This finding, together with prior literature, could imply that prospective outcome retrieval may represent a universal mechanism underlying decision-making. Nevertheless, the circumstances under which outcome retrieval manifests and its links to deliberation demands are under-studied.

Our results further show that in decisions that require deliberation, the strength of outcome retrieval could explain the capacity of participants to select the likely outcome. In context-free decisions, where the best choice was not altered by decision context, there was a significantly stronger than chance outcome retrieval, but this was not related to behavioral performance. We showed that the lack of links to behavior in context-free decisions was not due to a ceiling effect in participants’ performance, by repeating the same analysis for the first and second half of the experiment (Fig. S11). Moreover, we showed that the neuro–behavioral coupling that we observed for context-dependent decisions was specific to the most likely outcome as there was no coupling between context-dependent behavioral performance and retrieval of the context-free outcome.

Although our results clearly show that retrieval of neural representations during deliberation is linked to the probability of selecting the most likely outcome, the exact mechanism underpinning this finding is unknown. To this aim, we conducted an exploratory analysis which showed that how fast the behavioral performance improves can be explained by the change in the strength of outcome retrieval for context-dependent but not context-free decisions. This finding was specific to decisions where context can alter the decision outcome, consistent with the neuro–behavioral coupling observed at an aggregate level. In the literature, it has been shown that memory retrieval during rest can help consolidate newly acquired experience (Carr et al. 2011; Pfeiffer and Foster 2013; Ólafsdóttir et al. 2018; Schapiro et al. 2018). Our findings indicate that outcome retrieval could be a proxy for learning, especially in nontrivial cases where contextual information can alter decision outcome. We are adding to this literature by showing that in humans learning through retrieval of neural representations takes place not only in “passive” off-task rest (Schapiro et al. 2018) and sleep (Schönauer et al. 2017; Belal et al. 2018; Zhang et al. 2018) but also while “actively” decisions are made. Further research is needed to uncover the exact mechanism behind this which could help improve our understanding of learning disorders.

Previous studies which have reported that retrieval of prospective outcome is mediating behavioral choices, have either used complex sequences that unfold over several seconds and require intensive planning (Wise et al. 2021; Wimmer et al. 2023), or have changed the outcome of choices over the course of the experiment (Doll et al. 2015; Wise et al. 2021; Wimmer et al. 2023). These demanding factors naturally increase the need for deliberation. In our task, we directly manipulated deliberation demands by including decisions whose outcome can be altered by context, and decisions whose likely outcome is unaltered. Context-dependent decisions exhibit different behavioral readouts compared to context-free ones, namely longer reaction times and lower decision accuracy (Fig. 1D). This already suggests that context-free decisions may be mediated by different neural computations than context-dependent ones. Our findings confirm this view at a behavioral level. At a neural level, we showed that outcome retrieval may take place for both types of decisions, but that it is behaviorally relevant only for context-dependent ones. One interpretation of our finding is that the neural calculations underlying outcome retrieval are computationally expensive and are therefore not used for decisions that do not require long deliberation. An example of decisions with low deliberation demands is habitual decisions. Here, we do not explicitly study those decisions, although we cannot exclude that in the late phases of the experiment, when participants had fully learned the context-object-outcome associations, they might make more “automatic” decisions (Wang et al. 2022). However, we excluded the possibility that our findings on neuro–behavioral coupling and their context-dependence were simply reflecting mechanisms underlying “automatic” decisions, by repeating our analyses for the first and second half of the experiment. In the first half, the object-context-outcome associations were still clearly being learnt, as indicated by longer reaction times and lower behavioral performance (Fig. S11a, Table S1). For the complex process of decision-making, a network of brain regions have been reported to mediate retrieval of neural representations, encompassing key areas such as the frontal cortex, known for encoding value (Doll et al. 2015; Castegnetti et al. 2020; Minxha et al. 2020; Wise et al. 2021), along with the visual cortex (Wimmer and Shohamy 2012; Castegnetti et al. 2020) and hippocampus (Wimmer and Shohamy 2012; Wise et al. 2021). In our study, we observed retrieval of outcome representations which were extracted from topographic EEG responses without any spatial restriction (Castegnetti et al. 2020; Wise et al. 2021). In this work, we did not conduct source localization as our main goal was to investigate the temporal structure of context-dependent outcome retrieval and not its spatial correlates. Future work can link the two, ideally based on intracranial EEG which offers the advantage of high spatial resolution (Johnson et al. 2020).

Neural retrieval has been linked to hippocampal theta oscillations in both animal (Jadhav et al. 2012; Wu et al. 2017; Wang et al. 2020) and human studies (Osipova et al. 2006; Kerrén et al. 2018; ter Wal et al. 2021), particularly in the context of spatial navigation. This raises the question of whether the frequency of outcome retrieval observed in our study reflects theta oscillations. While there is some overlap between the theta frequency range (4 to 8 Hz) and the range in which outcome retrieval exceeds chance levels (4.5 to 19.1 Hz), our findings span a broader spectrum that includes alpha and beta oscillations as well. Therefore, we cannot conclude that the observed outcome retrieval is solely driven by theta oscillations. Furthermore, much of the existing literature focuses on spatial navigation and the activity of hippocampal place cells (Jadhav et al. 2012; Wu et al. 2017; Wang et al. 2020) which encode spatial information and may not fully account for neural activity patterns at the whole-brain level. Future research should investigate how outcome retrieval is implemented and communicated through among regions that play a role in neural outcome retrieval.

What is represented in the brain is not static such that factors like context can change these representations (Castegnetti et al. 2021; Hahamy et al. 2023; Muhle-Karbe et al. 2023). The effect of context on neural representations has been shown in memory tasks (Bornstein and Norman 2017; de Bettencourt et al. 2019) as well as spatial navigation (Muhle-Karbe et al. 2023) and goal-directed decision-making (Frömer et al. 2019; Castegnetti et al. 2021; Moneta et al. 2023). To this, we add that context modifies not only value computations but that it is also dynamically integrated into outcome retrieval. Although there is consensus that vmPFC is the region encoding and tracking decision contexts, the temporal dynamics of context integration into the decision process are unknown. Here, we leveraged the high temporal resolution of EEG, to show that context was integrated very early into the decision deliberation, starting already at 0.16 s after context is revealed. Notably, context strengthened the retrieval of relevant outcomes. Our findings for early integration of context into a decision provide neural evidence to recent behavioral and eye-tracking findings which show that context-relevant information is prioritized in information gathering from the beginning of the deliberation process (Sepulveda et al. 2020). Here, we additionally show that context is not only altering valuation but also the neural implementation of prospective outcome retrieval itself.

In summary, our study provides evidence supporting prospective outcome retrieval as a mechanism underlying choice deliberation in the brain. Importantly, our findings demonstrate the pivotal role of context in decision-making which affects both the deliberation process and consequent behavioral outputs. This emphasizes the necessity of including context as a crucial variable in future investigations of outcome retrieval, especially to simulate real-world scenarios where context can alter meaning and shape decision outcomes. Context may be contributing to decision uncertainty by modulating the choice and outcome. Therefore, the observed robust outcome retrieval during deliberation in context-dependent decisions might arise from a more global mechanism for decision-making under uncertainty. These insights contribute to a broader understanding of decision-making processes and have implications for future research and theoretical frameworks in cognitive neuroscience.

## Supplementary Material

OutcomeRepresentation-Supplementary_Material_FINAL_bhae483

## Data Availability

The data used in this study can be provided by the corresponding author upon reasonable request. The code for the analysis is available on https://github.com/CCNBern/OutcomeRepresentation.
