# Supplementary material for: Context changes retrieval of prospective outcomes during decision deliberation
Source: Cereb Cortex. 2024 Dec 22;34(12):bhae483. doi: 10.1093/cercor/bhae483 (PMC11663511; doi:10.1093/cercor/bhae483)
Supplement: OutcomeRepresentation-Supplementary_Material_FINAL_bhae483 [file outcomerepresentation-supplementary_material_final_bhae483.docx]

**Supplementary Information** **
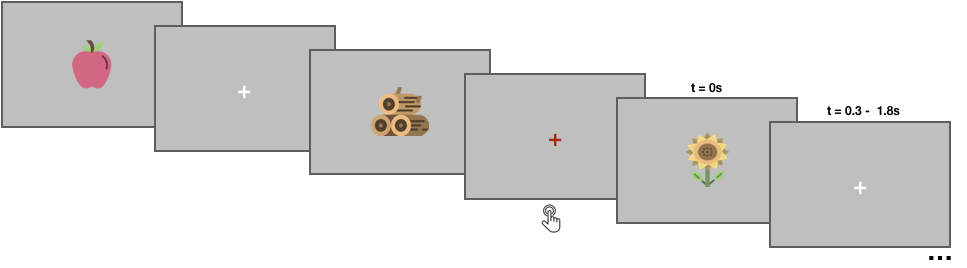
**

**Fig. S1: Design of functional localiser.** Participants were presented with an outcome image for 0.3 s, followed by a fixation cross, presented for 1.5 - 2.5 s. The order of the presented images was random. To ensure that participants were attentive during the functional localiser, they were asked to press a button when a red fixation cross appeared.


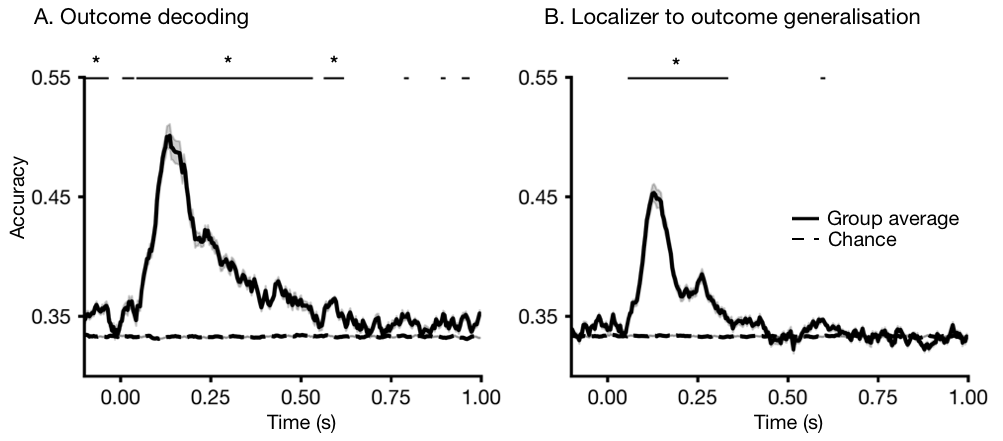


**Fig. S2: Decoding identity from outcome period and generalisation of identity decoding from localizer to outcome period.** (A) Decoding of outcome identity during outcome presentation after the decision in the decision-making task (black solid line) vs. chance (black dashed line). Classification accuracy was significantly above chance level (p_FDR_<0.05) from 0.05 s to 0.53 s and peaked at 0.14 s with a peak accuracy equal to 0.50 ± 0.01 across participants. (B) Generalisation of trained models from the functional localizer to the outcome presentation period (black solid line) vs. chance (black dashed line). Classification performance was significantly above chance (p_FDR_ < 0.05) from 0.06 s to 0.33 s, and peaked at 0.13 s with 0.45 ± 0.01 accuracy, confirming the generalisation of EEG patterns from the functional localizer to the outcome period in the decision-making task.


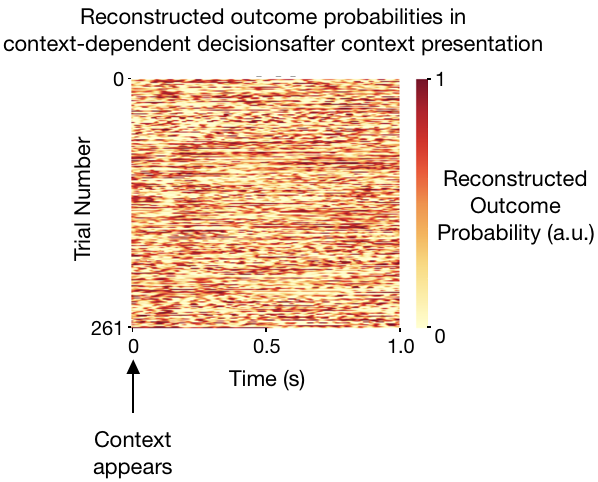


**Fig. S3: Exemplar time course of reconstructed probabilities for the most likely outcome across trials for context-dependent condition after context presentation.**


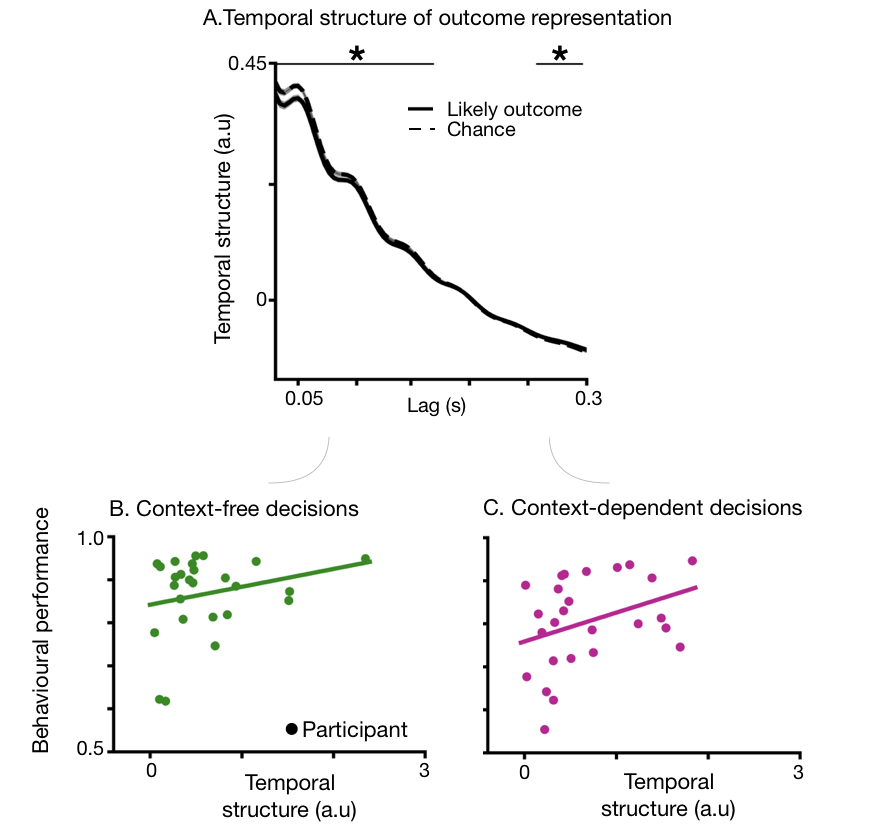


**Fig. S4: Temporal autocorrelation of reconstructed outcome.** (A) To compute the temporal structure of prospective outcome retrieval, we computed its temporal autocorrelation, which quantifies how similar a signal is to its future values (Castegnetti et al. 2020). The temporal autocorrelation of reconstructed outcome representation was significantly different from chance for time lags between 0.031 and 0.168 s, and between 0.259 and 0.297 s (Wilcoxon signed-rank test, p_FDR_ < 0.05). For positive autocorrelation values the likely outcome was retrieved in a significantly different from chance manner, but lower than chance levels. This may seem counter-intuitive at first, especially considering studies with only two prospective outcomes (Castegnetti et al. 2020). Nevertheless, this finding suggests that prospective outcome may be retrieved in a non-continuous manner, as previously suggested (Wise et al. 2021; Kurth-Nelson et al. 2016; Liu et al. 2019). Chance level by contrast fluctuates around 0.33 (theoretical chance, Figure 3B), which results in a stronger autocorrelation (i.e. temporal stability) than the one corresponding to the actual likely outcome. These findings support our hypothesis that likely outcomes are retrieved in the brain during decision deliberation, before the outcome itself is experienced. (B-C) Neuro-behavioural coupling between the behavioural performance and temporal autocorrelation of outcome retrieval in (B) context-free and (C) context-dependent conditions. For context-free decisions, the regression was not significant (panel B, F(1,24) = 1.48, p = 0.24) while for context-dependent, we found a strong positive regression however, it did not exceed the statistical threshold (panel C, F(1,24) = 3.24, p = 0.084).


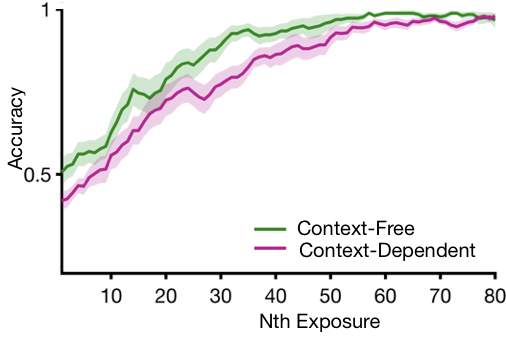


**Fig. S5: Trajectory of behavioural accuracy over number of (Nth) exposures to the context-free (green) and contest-dependent (pink) object and context pairs.**


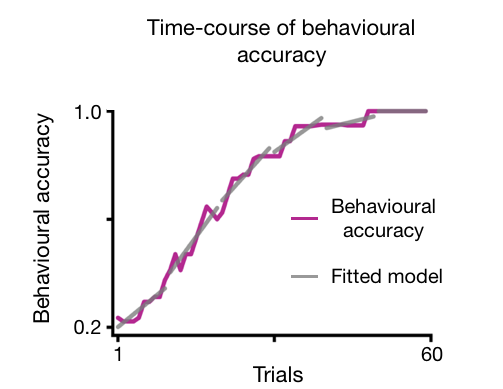


**Fig. S6: Exemplar time course of behavioural accuracy across trials from one participant.** The behavioural accuracy was computed in a sliding window fashion (N = 20 trials) over the course of the experiment (in pink). Then linear models were fitted to every 10 trials to quantify the changes in behaviour (in grey). The same approach was followed for computing the time course of retrieval strength for each object/context/outcome association per participant.


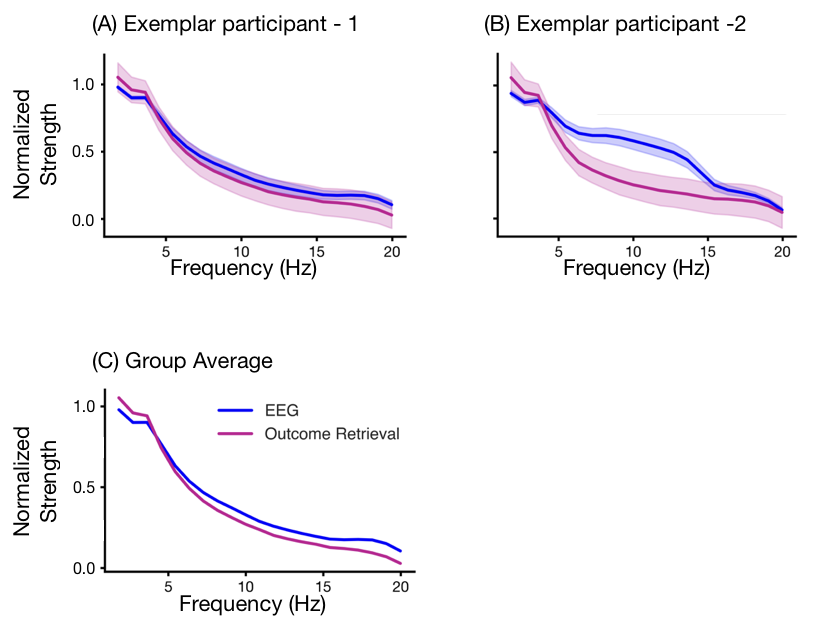


**Fig. S7: PSDs of outcome retrieval (pink) and of EEG data (blue).** (A-B) Average across trials (thick lines) and standard deviation across trials (shaded areas) for exemplar participants. At the single participant level, the overall distribution of power in the PSDs of outcome retrieval vs. preprocessed EEG data was similar for some participants (A), while for others not (B), making links between individual PSDs and EEG traces not that straightforward. (C) The group average (thick lines) and standard deviation across participants (shaded areas). At the group level, where inter-individual differences are eliminated, the distribution of power in the PSDs of outcome retrieval vs. EEG data was largely similar. This is due to the fact that the temporal dynamics of the reconstructed probabilities of outcome retrieval are, to some extent, dictated by the underlying EEG dynamics, and therefore follow similar temporal patterns. To ensure that our findings do not merely reflect modulations of oscillatory power on the EEG data, but modulations of outcome retrieval, in our analyses we compared the deviation of PSDs of true reconstructed probabilities for the likely outcome with chance ones (Fig. 4A).


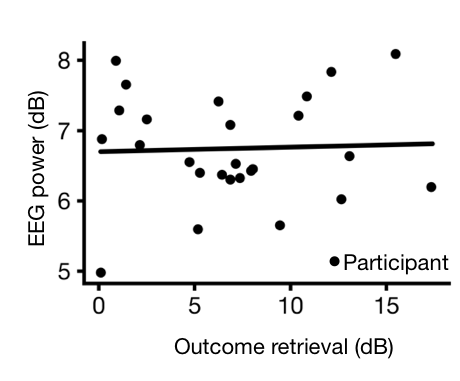


**Fig. S8: Control analysis for a possible link between PSD of outcome retrieval and of EEG.** We computed the PSD of single-trial EEG responses and summed the power across those frequencies (4.5-19.1 Hz) where the PSD of outcome retrieval was significantly different from chance (see Fig. 4). We then regressed the summed power from PSD of EEG on the strength of outcome retrieval. The regression was not significant (F(1,24) = 0.039, p = 0.85), indicating that the reported PSD of outcome retrieval does not simply reflect oscillatory activity in the EEG. Each dot corresponds to a participant.


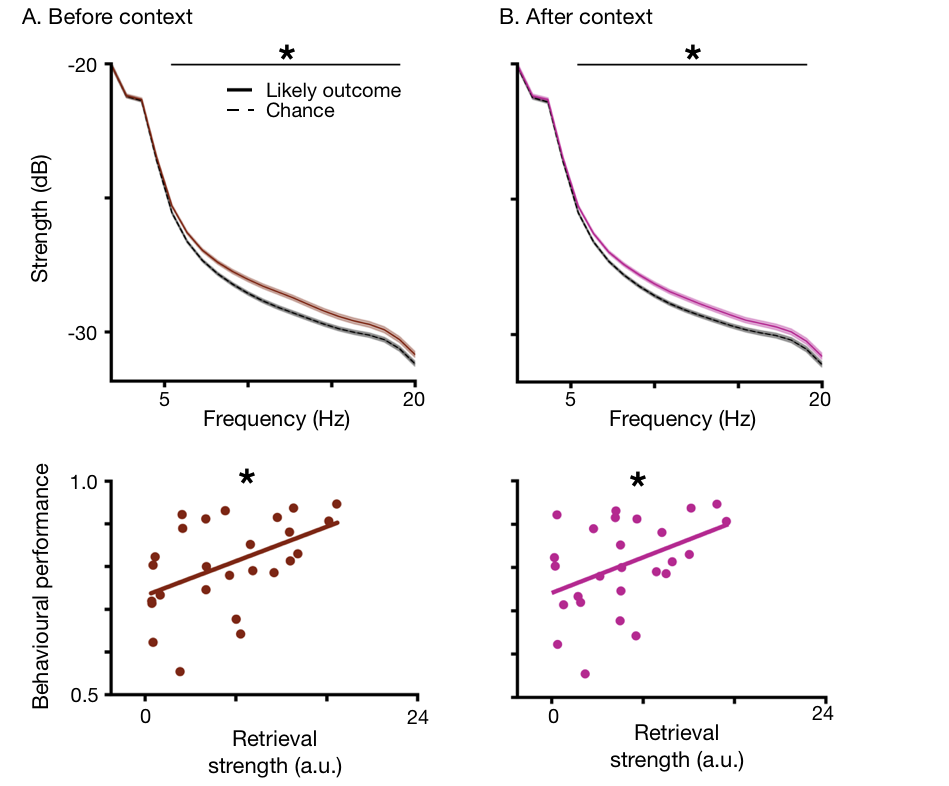


**Fig. S9:** **Outcome retrieval and its relation with behavioural performance in context-dependent decisions (A) before and (B) after context presentation.** (Top) Strength of outcome retrieval (solid line in brown in A and in pink in B) vs. chance (dashed black line), quantified via power spectral analysis on reconstructed outcome probabilities. Chance level was evaluated based on the randomly permuted classifiers of outcome images. Horizontal bar with asterisks (*) indicate frequencies which were different from chance. (Bottom) Neuro-behavioural coupling between the behavioural performance and strength of outcome retrieval in (A) before and (B) after the presentation of context. Regression of behavioural performance on strength of outcome retrieval both before (panel A, bottom, linear regression, F(1, 24) = 7.5, p = 0.011) and after (panel B, bottom, linear regression, F(1, 24) = 5.39, p = 0.03) was statistically significant.


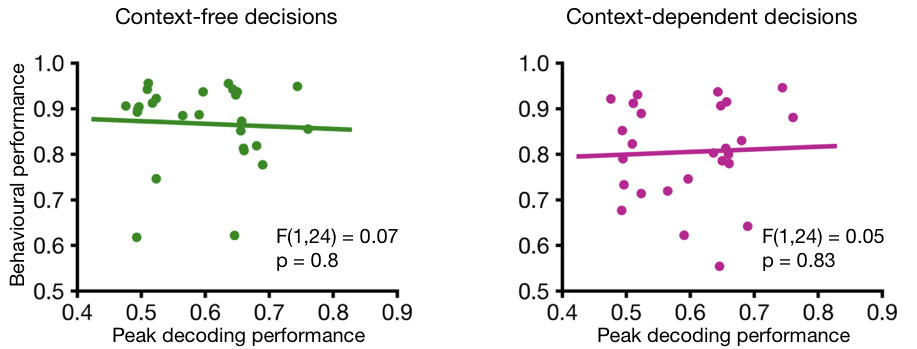


**Fig S10: Regression of behavioural performance on peak decoding performance.** The regression was not significant for neither context-free (left panel, green, linear regression, F(1, 24) = 0.07, p = 0.8) nor context-dependent decisions (right panel, pink, linear regression, F(1, 24) = 0.05, p = 0.83).


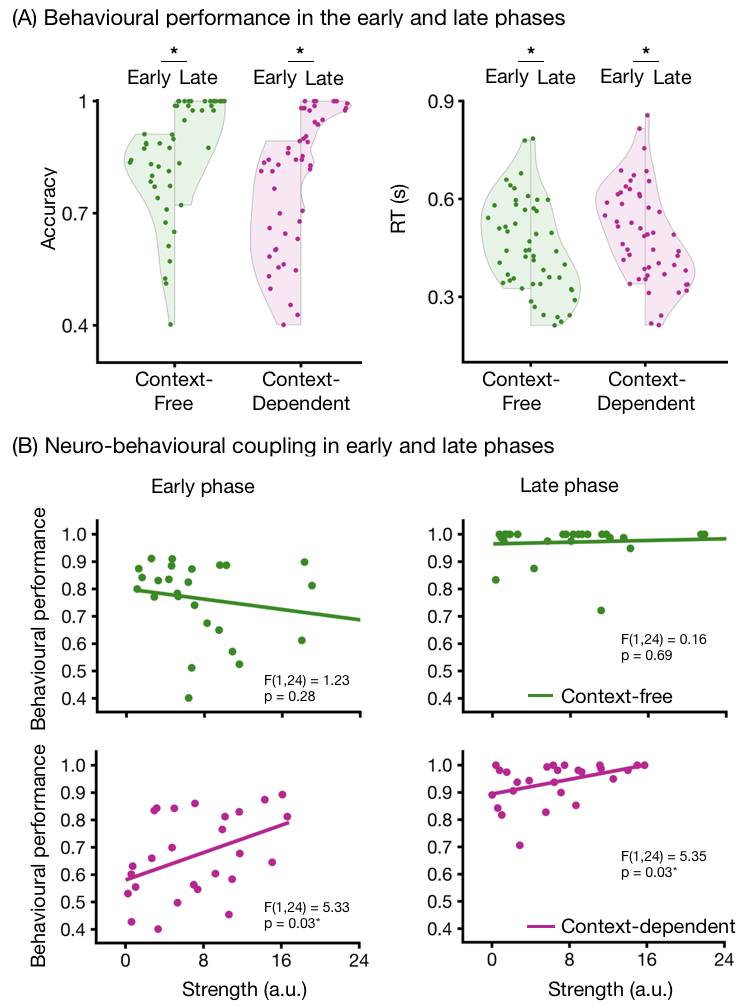


**Fig S11: Behavioural performance and neuro-behavioural coupling in early and late phases.** (A) Average behavioural accuracy (left) and response time (right) for context-free (green) and context-dependent (pink) decisions in the early vs. late phase of the experiment. Participants responded faster in the late than in the early phase of the experiment for both context-free (green, linear regression, F(1,50) = 48.14, p = 7.5x10^-9^) and context-dependent decisions (pink, linear regression, F(1,50) = 64.22, p = 1.6x10^-10^). Moreover, participants’ responses were more accurate in the late than in the early phase for both context-free (green, linear regression, F(1,50) = 10.56, p = 0.002) and context-dependent decisions (pink, linear regression, F(1,50) = 11.92, p = 0.001). Details of the models are provided in Table 1 below. (B) Regression of behavioural performance on the strength of outcome retrieval in context-free (green) and context-dependent (pink) conditions in early (left) and late (right) phases of the experiment. The regression was significant only for context-dependent decisions in both early (left, pink, linear regression, F(1, 24) = 5.33, p = 0.03) and late phase (right, pink, linear regression, F(1, 24) = 5.35, p = 0.03).


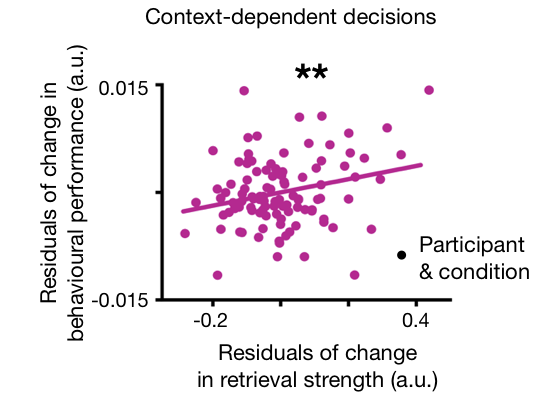


**Fig. S12:** **Neuro-behavioural coupling between the change in behavioural accuracy and in strength of outcome retrieval in context-dependent decisions after controlling for average behavioural performance by adding average behavioural performance as an additional predictor.** For context-dependent decisions, change in outcome retrieval (F(1,101) = 6.89, p = 0.01) remained significant and average behavioural performance was also significant (F(1,101) = 44.75, p < 0.001). For context-free ones, average behavioural performance was significant (F(1,49) = 66.67, p < 0.001) however, the change in outcome retrieval (F(1,49) = 3.96, p = 0.052) was not significant as in the original analysis (Fig. 5). To visualize the significant main effect of change in outcome retrieval in context-dependent decisions, we conducted a partial regression by regressing the residuals of change in outcome retrieval strength (after controlling for average behavioural performance and random factor of participant) on the residuals of change in behavioural performance. This partial regression yielded a significant positive relationship (F(1,102) = 6.9, p = 0.01), reinforcing the main findings of the mixed-effects model. Each data point corresponds to a pair of object and context for a participant.


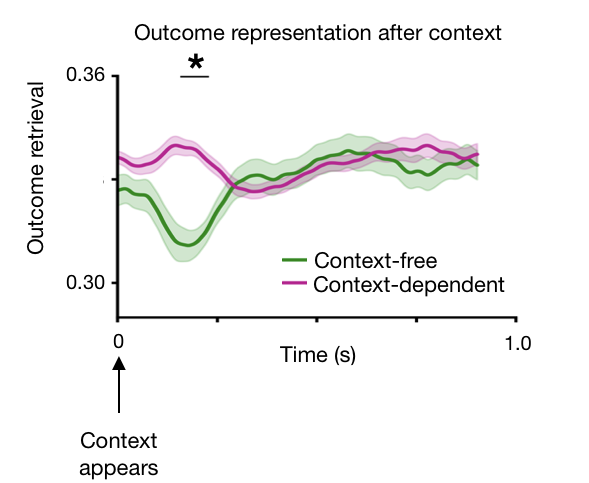


**Fig. S13: Reconstructed outcome probabilities without binarization in context-free (green) and context-dependent (pink) decisions after the presentation of context.** Without binarization, we found a significant difference between reconstructed outcome probabilities in context-dependent and context-dependent decisions between 0.16 s and 0.23 s post-context onset (Wilcoxon signed-rank test, p_uncorr_ < 0.05, the same time range reported in Fig. 6A).

**Table S1*. Results of regression on behavioural accuracy (model-1) and reaction time (model-2)***

|  | Full model | | | Experiment phase | | Context dependence | | Interaction | |
| --- | --- | --- | --- | --- | --- | --- | --- | --- | --- |
|  | DF | F | p | t | p | t | p | t | p |
| Model-1: Accuracy | 3, 100 | 40.51 | <2x10^-16^*** | 6.61 | 1.9x10^-9^*** | -2.84 | 0.005** | 1.25 | 0.21 |
| Post-hoc: Context-free | 1, 50 | 48.14 | 7.5x10^-9^*** | 6.94 | 7.5x10^-9^*** | - | - | - | - |
| Post-hoc: Context-dependent | 1, 50 | 64.22 | 1.6x10^-10^*** | 8.01 | 1.6x10^-10^*** | - | - | - | - |
| Model-2: RT | 3, 100 | 8.24 | 5.95x10^-5^*** | -3.2 | 0.002** | 1.20 | 0.23 | -0.21 | 0.83 |
| Post-hoc: Context-free | 1, 50 | 10.56 | 0.002** | 3.25 | 0.002** | - | - | - | - |
| Post-hoc: Context-dependent | 1, 50 | 11.92 | 0.001** | 3.45 | 0.001** | - | - | - | - |
